# Phylogenetic conservation of substrate use specialization in leaf litter bacteria

**DOI:** 10.1371/journal.pone.0174472

**Published:** 2017-03-30

**Authors:** Kristin L. Dolan, Jeniffer Peña, Steven D. Allison, Jennifer B. H. Martiny

**Affiliations:** Department of Ecology and Evolutionary Biology, University of California-Irvine, Irvine, California, United States of America; University of Massachusetts, UNITED STATES

## Abstract

Environmental change will influence the ecosystem processes regulated by microbial communities, including leaf litter decomposition. To assess how microbial communities and their functioning might respond to increases in temperature, we quantified the distribution of traits related to carbon substrate utilization and temperature sensitivity in leaf litter bacteria isolated from a natural grassland ecosystem in Southern California. The isolates varied substantially in their carbon substrate use, as well as their response to temperature change. To better predict the functioning and responses in natural communities, we also examined if the functional and response traits were phylogenetically patterned or correlated with one another. We found that the distribution of functional traits displayed a phylogenetic pattern, but the sensitivity of the traits to changes in temperature did not. We also did not detect any correlations between carbon substrate use and sensitivity to changes in temperature. Together, these results suggest that information about microbial composition may provide insights to predicting ecosystem function under one temperature, but that these relationships may not hold under new temperature conditions.

## Introduction

Over the next fifty years, southern California is expected to experience a range of environmental changes, including increased temperature, nitrogen deposition, and precipitation variability [1]. While much research has been devoted to predicting how plant communities will respond to these environmental changes, the response of microbial communities remains relatively unknown (but see [2–4]). Microbial communities play a central role in many ecosystem processes, including the terrestrial carbon cycle through mediation of leaf litter decomposition. Indeed, decomposition is the largest driver of carbon transfer from the biosphere to the atmosphere [5] and is performed almost exclusively by fungi and bacteria [6]. Therefore, determining how microbial communities will respond to changing environmental conditions may improve predictions of future ecosystem carbon cycling.

One approach to linking microbial composition, ecosystem functioning, and environmental change is to identify traits that influence the structure and functioning of microbial communities [7–10]. Plant ecologists have proposed dividing such traits into two, non-exclusive types: response and effect traits [11]. Response traits determine how an organism’s abundance changes in the face of new environmental conditions. For example, the physiological response of microorganisms to increased temperature has been well documented, and includes elevated rates of enzymatic reactions and respiration (reviewed in [12]), both of which influence decomposition rates. In contrast, effect traits (hereafter “functional” traits as in [13]) are characteristics that influence ecosystem properties or processes such as nutrient cycling and trace gas emissions. Together, knowledge about the response and functional traits of all species in a community may help predict how composition and functioning will shift in response to environmental change [11, 14–18].

For this study, we characterized functional and response traits of bacterial taxa isolated from natural leaf litter communities located in southern California [19]. Bacteria dominate these communities [20], and their composition varies seasonally and in response to environmental change, including drought and nitrogen addition [21]. Furthermore, variation in the microbial composition of these litter communities has been shown to impact the decomposition rate of leaf litter [22, 23], as well as the response of ecosystem process rates to environmental change [24]. Despite these results, the mechanisms underlying why litter bacteria respond to environmental change, and why their composition influences decomposition rates, remain unknown. A quantitative understanding of the functional and response traits of this bacterial community might help to elucidate these mechanisms.

In addition to examining the traits of specific organisms, phylogenetic relatedness of community members could potentially be used to extrapolate trait distributions to the broader community (reviewed in [25, 26]). In particular, more closely related species might, due to sharing a recent common ancestor, possess a more similar suite of traits. Indeed, a number of studies on microbes [27, 28] and plants [29] have shown that the phylogenetic structure of a community is correlated with functioning, although this is not always the case [30]. In addition, the vast majority of carbon-use traits in Bacteria and Archaea appear to be non-randomly distributed, although the depth at which these traits are conserved varies [31]. Response traits can also be patterned by phylogeny, such that perturbed communities consist of more closely related species than would be expected by chance [32, 33]. If functional and response traits in leaf litter bacteria are phylogenetically structured, the composition of a community might help estimate the ecosystem’s decomposition rate under varying environmental conditions. In addition to identifying the phylogenetic patterning of traits, correlations between functional and response traits across organisms could also be used to extrapolate trait distributions and predict future process rates.

**Fig 1:**
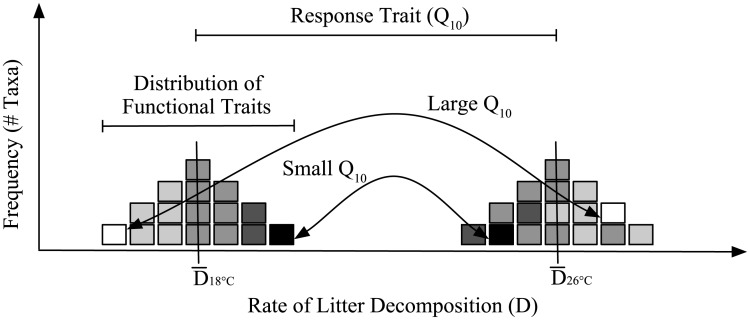
Theoretical distribution of functional and response traits. Theoretical distribution of one functional trait, litter decomposition rate (D), and one response trait, temperature sensitivity (Q_10_), for 16 bacterial taxa (each represented by a single square). As seen here, the darkest square, representing a single bacterial taxon, is the best litter degrader at 18°C, but its rate does not shift dramatically at 26°C (small Q_10_). In contrast, the taxon represented by the lightest square is the poorest litter degrader at 18°C but its rate increases substantially at 26°C (large Q_10_). In this case there is a correlation between functional and response traits, such that better litter degraders at 18°C are, on average, less sensitive to changes in temperature.

To determine the distribution of physiological traits in leaf litter microbial communities (Fig 1), we used physiological and metabolic assays to measure substrate use traits in 16 leaf litter bacteria isolates. The bacteria were isolated from a complex grassland leaf litter community; many belong to taxonomic groups that are abundant in whole community sequence data [21]. The assays were repeated along a temperature gradient, yielding functional traits distributions at three different temperatures. By comparing trait values across temperatures, we estimated the temperature response (Q_10_) of each substrate use (functional) trait for each isolate.

We then tested if functional and response traits were phylogenetically conserved. We hypothesized that, like other microorganisms, temperature response of leaf litter bacteria would be more phylogenetically conserved than their functional, substrate use traits [31, 34, 35]. The use of particular carbon substrates is a simple trait that can be enabled by the presence of just one or two genes. Thus, the ability to use these substrates has likely evolved many times, either by lateral transfer or point mutation, and is therefore generally finely conserved amongst bacteria [35]. In contrast, the optimal temperature for bacterial growth is presumably determined by a variety of genetic factors.

Lastly, we tested whether the functional and response traits were correlated with one another; a correlation between these traits would suggest a tradeoff between substrate use and temperature sensitivity. Many traits are correlated with one another, especially when physiological trade-offs constrain trait values [36, 37]. We hypothesized that these physiological tradeoffs also exist in leaf litter bacteria. For instance, we expected that taxa that utilize a larger set of substrates (i.e., more generalist life-strategies) would be less sensitive to changes in temperature. Likewise, taxa that utilized fewer substrates (i.e. specialist life-strategies) would be more sensitive to temperature changes.

## Methods

### Strain isolation and identification

The bacterial isolates used in this study were isolated from leaf litter collected from the Loma Ridge research site [19] located 5 km north of Irvine, California, USA (33 44’N, 117 42’W, 365 m elevation) in January, April, July, and September of 2011. During this period, the site was dominated by the annual grass genera *Avena*, *Bromus*, and *Lolium* the annual forb genera *Erodium* and *Lupinus* and the native perennial grass *Nassella pulchra*. The temperatures in this system are moderate, with an average winter (November through April) high of 21.0°C and low of 6.2°C and an average summer (May through October) high of 27.3°C and low of 13.1°C; the mean annual temperature is 17°C.

Once collected, the litter was ground and then washed with sterilized deionized (DI) water. Litter fragments (106–212 μm) were suspended in sterile DI water and the resulting solution was passed through a 100 μm filter (EMD Millipore, Billerica MA), and 20 ml of the filtered litter solution was mixed with 20 ml of 0.6% carboxymethylcellulose solution (an emulsifier). This slurry was then plated onto Luria Broth (LB) agar plates or litter agar plates and allowed to incubate at 21°C for up to two weeks (as described in [24]). Individual colonies were transferred to fresh LB agar plates at least three times to purify the cultures. Prior to the experiments, the cultures were stored at -80°C in a glycerol solution (52% glycerol, 2% MgSO4, and 2% Tris-Cl; pH = 7.6).

We identified each bacterial isolate by PCR amplification and Sanger sequencing of the 16S rRNA gene. DNA extract from each isolate was added to a PCR cocktail containing 1.5 units of HotMasterTaq polymerase (5PRIME, Gaithersburg MD) 1x PreMixF (FailSafe), 0.3 μM of each primer (pA: 5'-AGAGTTTGATCCTGGCTCAG-3' and pH: 5'-AAGGAGGTGATCCAGCCGCA-3' [38]), and H_2_O to a final volume of 31 μl. Following an initial denaturation step at 95°C for 4 min, PCR was cycled 30 times at 95°C for 40 sec, 55.5°C for 30 sec, 72°C for 2 min, and a final extension at 72°C for 3 min 30 sec. All PCRs here and below were performed on a PTC-100 thermocycler (Bio-Rad, Hercules CA). The taxonomic identity of each isolate was determined by matching our sequences to the most closely related cultured representative using the BLAST tool in the GenBank database. We further confirmed the taxonomic identities of the isolates using the Ribosomal Database Project (RDP) Naive Bayesian rRNA Classifier (Version 2.11) [39]. We aligned the 16S rRNA sequences using the SINA aligner (www.arb-silva.de/ [40]). A maximum likelihood tree was estimated with 100 bootstrap replications using a transition/transversion ratio = 2, a constant base rate variation among sites, and empirical base frequencies using PHYLIP v. 3.68 [41]. A total of 16 isolates were selected for this study because 1) they had already been taxonomically identified and characterized in another study [42], 2) they provided a range of phylogenetic distances, and 3) many belonged to taxonomic groups common in leaf litter bacterial communities [21]. Indeed, in a PCR survey of litter from the same ecosystem [21] found the relative abundance of the genus’s represented in this study ranged from 0–18.67% of the samples. A list of the isolates used in this study, including the relative abundance of their respective genus in natural communities, is shown in Table 1.

**Table 1:** Bacterial isolates used in this study.

Taxonomic Identification	Accession Number	Natural Abundance of Genus (%)
**Proteobacteria**		
*Pseudomonas fluorescens*	KF733338	<0.01
*Pseudomonas synxntha*	KF733328	
*Pseudomonas sp*. (isolate 1)	KF733329	
*Pseudomonas sp*. (isolate 2)	KF733330	
*Erwinia billingiae*	KF733325	<0.01
*Duganella zoogloeoides* (isolate 1)	KF733339	NA (4.00)
*Duganella zoogloeoides* (isolate 2)	KF733333	
**Actinobacteria**		
*Sanguibacter sp*.	KF733318	0.29
*Frigoribacterium sp*.	KF733312	15.34
*Schumannella luteola*	KF733311	NA (36.77)
*Microbacterium sp*.	KF733319	0.01
*Curtobacterium sp*.	KF733315	18.67
*Curtobacterium flaccumfaciens*	KF733308	
**Bacteriodetes**		
*Pedobacter borealis*	KF733323	3.15
*Flavobacterium sp*.	KF733322	0.78
*Chryseobacterium sp*.	KF733320	0.32

The 16 bacterial isolates used in this study, and their corresponding GenBank accession number. If the bacterial genus was detected in natural litter pyrosequencing data [21], the genus’ relative abundance ("Natural Abundance”) is also shown. If no sequences belonging to the genus were identified, the relative abundance of the family is given in parentheses.

### Simple carbon use assay

Approximately 10 days before each assay, glycerol stocks of 16 bacterial isolates were removed from -80°C and streaked onto LB agar plates. Plates were incubated at room temperatures (roughly 23°C) for approximately 6 days or until single colonies appeared. Single colonies were picked from each culture and inoculated into 25 ml of LB media. To allow for temperature acclimation, liquid cultures were grown with agitation (100 rpm) at each experimental temperature (18°C, 22°C, or 26°C) for 5 days and transferred at least once to new media. On the day of inoculation, each culture was transferred to a 50 ml conical centrifuge tube and centrifuged at 4500 rpm for 10 minutes. The supernatant was discarded and the pellet was re-suspended in sterile saline solution (0.9% NaCl). This washing process was repeated a total of four times to remove residual LB media.

**Fig 2:**
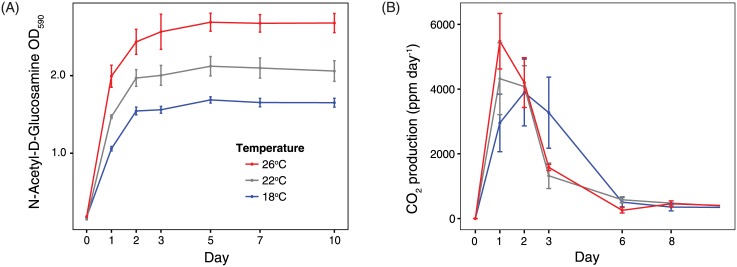
Sample EcoPlate and CO_2_ production data. The effect of temperature on *Erwinia billingiae’s* (A) ability to utilize the EcoPlate substrate N-acetyl-D-glucosamine and (B) mineralize leaf litter C. Each value plotted is mean (± SEM) based on three replicates.

After washing, cultures were diluted in saline solution to achieve an optical density at 600 nm (OD_600_) of 0.100 ± 0.05, and 100 μl of the diluted cell suspension was inoculated into each well of an EcoPlate (Biolog, Hayward CA). EcoPlates contain 31 unique substrates in triplicate plus 3 carbon-free controls controls in a 96 well-plate format. Through substrate consumption and consequent cellular respiration, an indicator dye is reduced and a purple pigment is produced. After inoculation, plates were incubated at the same temperature used during acclimation (18°C, 22°C, or 26°C) for ten days. Substrate utilization (OD_590_) and bacterial growth (turbidity; OD_750_) were assessed at time 0, and 1, 2, 3, 5, 7, and 10 days using an automated Synergy 4 (BioTek, Winooski VT) microplate reader (Fig 2A). A substrate was considered utilized if the OD_590_ (mean based on three replicates) after 10 days was ≥0.30, which exceeded the maximum OD_590_ of the substrate-free controls (S1 Dataset). *Sanguibacter sp*. was removed from all EcoPlate analyses due to the development of pigment in negative control wells.

To estimate growth parameters, we fit the substrate utilization data to a modified Gompertz equation:

$$y=Aexp\left\{-exp\left[\frac{\mu_{max}e}{A}\left(\lambda -t\right)+1\right]\right\}+y_{0}$$

where *y* is cell density (or in this case mean OD_590_ of a particular substrate), *A* is the carrying capacity, *μ*_max_ is maximum growth rate, and *λ* is the lag time between inoculation and the initiation of exponential growth, and y_0_ is the initial inoculation density [43].

The response of bacterial growth to temperature was determined for every utilized substrate by calculating the Q_10_, the factor by which a biological process increases in rate for every 10°C change in temperature, based on *μ*_max_ values:

$$Q_{10-\mu_{max}=}{\left(\frac{\mu_{18{}^{\circ}C}}{\mu_{26{}^{\circ}C}}\right)}^{\left(\frac{10}{26{}^{\circ}C-18{}^{\circ}C}\right)}$$

where *μ*_18°C_ and *μ*_26°C_ are the estimated *μ*_max_ for the coldest (18°C) and warmest (26°C) incubation temperatures. To determine the sensitivity of substrate yield, Q_10-OD590_ values were calculated using the mean final OD_590_ value after 10 days. While most Q_10_ values were in the biologically relevant range, there were a few instances where a substrate was utilized at 26°C and not at 18°C. This produced an inflated Q_10_ value that is not easily comparable to the other Q_10_ data.

To test if the similarity of substrate use or temperature sensitivity was correlated with phylogenetic distance, we performed Mantel tests based on 999 permutations. Specifically, we compared the bacterial phylogenetic distance matrix with euclidean distance matrices of substrate usage (substrate use richness, mean OD_590_, or mean μ_max_) or temperature sensitivity (Q_10-μmax_ or Q_10-OD590_). The mean OD_590_ and mean μ_max_ represented the mean value across all three temperature treatments.

### Complex litter use experiment

Leaf litter (comprised of roughly 12% lignin, 48% cellulose, 33% hemicellulose, 4% crude protein, and 3% ethanol soluble carbohydrates [44] from the Loma Ridge site was collected in the summer of 2010, ground in a Wiley mill to pass a #20 mesh screen, and sterilized with roughly 23 kGy of gamma irradiation and autoclaving for 30 minutes. Sterilized litter (50 mg) was then added to each microcosm, an autoclaved glass vial (40 ml) with a sampling septum containing 4 g sterile sand. One culture (100 μl of the diluted cell suspension with 700 μl of saline solution) was slowly pipetted into each microcosm. Microcosms were placed in large plastic containers to keep humidity near 100% and stored at either 18°C, 22°C, or 26°C. Each isolate (n = 16) and temperature (n = 3) combination contained three replicate microcosms for a total of 144 microcosms.

CO_2_ concentrations in the microcosm headspace were measured on days 1, 2, 3, 6, 8, and 10 to estimate cumulative CO_2_ production, our metric for litter decomposition (Fig 2B; S1 Dataset). To keep the microcosms at atmospheric CO_2_ concentrations, the vial caps were kept loose except for 24 hours before each sampling. For each measurement, an 8 ml subsample of headspace gas was withdrawn by syringe and injected into an infrared gas analyzer (PP-Systems EGM-4). To determine the temperature responses of leaf litter decomposition, we compared the time required for the 40 ml vial to reach a concentration of 5000 ppm of CO_2_ at 18°C, 22°C, and 26°C, which corresponded to approximately 0.2% of litter carbon respired. Following an approach by Conant, Drijber, et al. [45], we estimated the Q_10_ of litter decomposition by dividing the time taken to respire 5000 ppm of CO_2_ at the coldest temperature (t_18°C_) by time taken at the warmest temperature (t_26°C_) and correcting for the actual incubation temperature differential (26°C-18°C):

$$Q_{10=}{\left(\frac{t_{18{}^{\circ}C}}{t_{26{}^{\circ}C}}\right)}^{\left(\frac{10}{26{}^{\circ}C-18{}^{\circ}C}\right)}$$

### Additional statistical analyses

Because the main effect of temperature on substrate usage and litter decomposition was non-linear, we used a mixed model ANOVA to analyze the effect of isolate, temperature, their interaction, and substrate (when applicable) on respiration, substrate use richness, OD_590_, and μ_max_. Isolate and substrate were treated as random factors and temperature as a fixed, categorical factor. We assessed the phylogenetic signal of functional and response traits among bacterial isolates using 999 permutations of Bloomberg’s K statistic as encoded in the R package ‘picante’. The input tree for all phylogenetic analyses was a maximum likelihood tree estimated from original alignment using the majority consensus tree of 100 bootstrap runs as a topological guide. To visualize general trends across the isolates, we organized the functional and response trait data into a single 7 trait x 15 isolate matrix (*Sanguibacter sp*. removed). Trait variables were normalized to a similar mean and variance, and Euclidean distances was calculated between all isolates. We then performed a Principal Component Analysis (PCA) and plotted the first two axes with a Pearson correlation overlay. The PCA identified 7 components of variance, which each explained between 1.1–43.6% of the total variance. The first two components, PCA1 and PCA2, accounted for 68% of total variance and are plotted in S4 Fig.

## Results

### Substrate use traits

**Fig 3:**
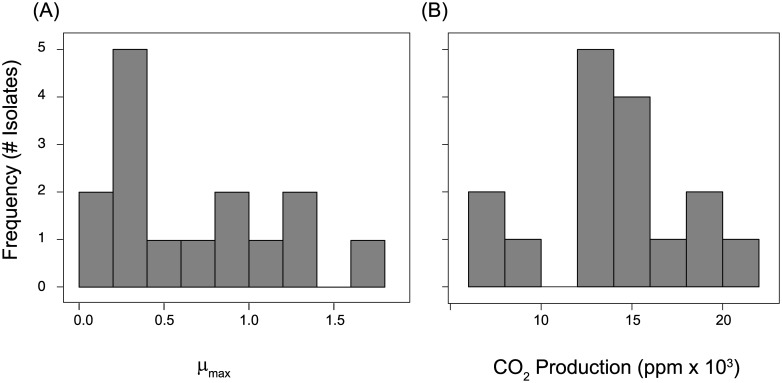
Distribution of functional traits. The distribution of two functional traits for all bacterial isolates: (A) maximum potential growth rate (μ_max_) on EcoPlate substrates, and (B) CO_2_ production from the mineralization of leaf litter C. All functional traits are based on the mean value across all temperatures and, when applicable, EcoPlate substrates.

Grassland litter bacteria varied greatly in their functional traits, as measured by their use of simple substrates and complex leaf litter (Fig 3). We first compared the ability of each isolate to use the 31 substrates on the EcoPlates (Table 2). All but two substrates, 2-hydroxy benzonic acid and phenylethylamine, were used by at least one isolate. At the intermediate temperature (22°C), the isolates used an average of 11.6 substrates. The breadth of this use ranged greatly, between 0 and 24 substrates depending on the isolate (S1 Fig). Two Actinobacteria isolates, *Schumannella luteola* and a *Frigoribacterium* sp., did not use any of the substrates (S1 Fig). The most commonly utilized substrates were the carboxylic acids pyruvic acid methyl ester, D-galacturonic acid, and glycyl-L-glutamic acid (Table 3).

**Table 2:** ANOVA results for functional traits.

	OD_590_			μ_max_			CO_2_ production		
Source of variation	df	Chisq	P	df	Chisq	P	df	Chisq	P
Substrate	1	162.2	<0.001	1	85.6	<0.001	NA	NA	NA
Isolate	1	33.6	<0.001	1	21.1	<0.001	1	15.3	<0.001
Temperature	2	12.7	0.002	2	13.6	0.001	1	10.1	0.006
Isolate*Temperature	1	7.9	0.005	1	0.4	0.6	1	7.1	0.008

Overall mixed-model ANOVA results for factors affecting substrate use (OD_590_), growth rate (μ_max_), and CO_2_ production rates. Substrate and Isolate terms are treated as random factors, while the Temperature term is treated as a fixed factor.

**Table 3:** Results for each Biolog EcoPlate substrate, including the number of isolates that utilized each substrate and Bloomberg K-statistics.

Biolog EcoPlate Substrate	Isolate Richness	OD_590_	μ_max_	Q_10-μmax_	Q_10-OD590_
**Amine**					
Phenylethylamine	0	NA	NA	NA	NA
Putrescine	6	0.11	0.08	0.07	0.07
**Amine Acid**					
L-Arginine	8	0.15	0.18	0.08	0.08
L-Asparagine	9	0.15	0.37**	0.07	0.07
L-Phenylalanine	1	0.39	0.53	0.53	0.53
L-Serine	9	0.66**	0.28*	0.08	0.24
L-Threonine	7	0.17	0.09	0.07	0.18
**Carbohydrate**					
D-Cellobiose	11	0.1	0.24	0.05	0.05
D-Galactonic Acid γ-Lactone	8	0.15	0.09	0.07	0.07
D-Mannitol	9	0.5**	0.24**	0.07	0.09
D-Xylose	11	0.07	0.07	0.12	0.49
D,L-α-Glycerol Phosphate	8	0.21*	0.25*	0.05	0.11
Glucose-1-Phosphate	9	0.1	0.14	0.05	0.06
i-Erythritol	5	0.1	0.09	0.14	0.13
N-Acetyl-D-Glucosamine	8	0.16	0.12	0.07	0.08
α-D-Lactose	7	0.03	0.14	0.04	0.36
β-Methyl-D-Glucoside	6	0.11	0.18	0.43*	0.08
**Carboxylic Acid**					
D-Galacturonic Acid	12	0.07	0.4*	0.07	0.09
D-Glucosaminic Acid	6	0.16	0.1	0.23	0.1
D-Malic Acid	8	0.08	0.1	0.01	0.01
Glycyl-L-Glutamic Acid	12	0.27*			0.07
Itaconic Acid	7	0.06	0.07	0.06	0.07
Pyruvic Acid Methyl Ester	12	0.08	0.18	0.23	0.1
α-Ketobutyric Acid	1	0.39*	0.53	0.53	0.53
γ-Hydroxybutyric Acid	4	0.07	0.07	0.06	0.06
**Phenolic Compound**					
2-Hydroxy Benzonic Acid	0	NA	NA	NA	NA
4-Hydroxy Benzonic Acid	5	0.11	0.11	0.05	0.06
**Polymer**					
Glycogen	5	0.02	0.02	0.44	0.51*
Tween 40	11	0.09	0.27	0.05	0.1
Tween 80	8	0.09	0.04	0.07	0.04
α-Cyclodextrin	2	0.42**	0.65**	0.63*	0.59*

The Bloomberg's K-statistic tests for a phylogenetic signal of average EcoPlate substate usage (OD_590_), growth rate (μ_max_), and temperature sensitivities of growth rate (Q_10-μmax_) and maximum (Q_10-OD590_). Asterisks (*) denote K-statistics that are statistically significant (* p-value ≤ .05; ** p-value ≤ .01).

Beyond binary use, we assayed the degree to which the isolates could use each substrate by estimating growth rate (μ_max_) and comparing a metric of cumulative substrate use (OD_590_ on day 10). Across all substrates, the isolates differed in both growth rate and cumulative substrate use (p<0.001; Table 2; Fig 3a). For instance, the ability of *Pseudomonas sp*. (isolate 1) to utilize pyruvic acid methyl ester at 26°C was 10x greater than *Pseudomonas synxntha* (μ_max_ = 4.67 and 0.467, respectively).

**Fig 4:**
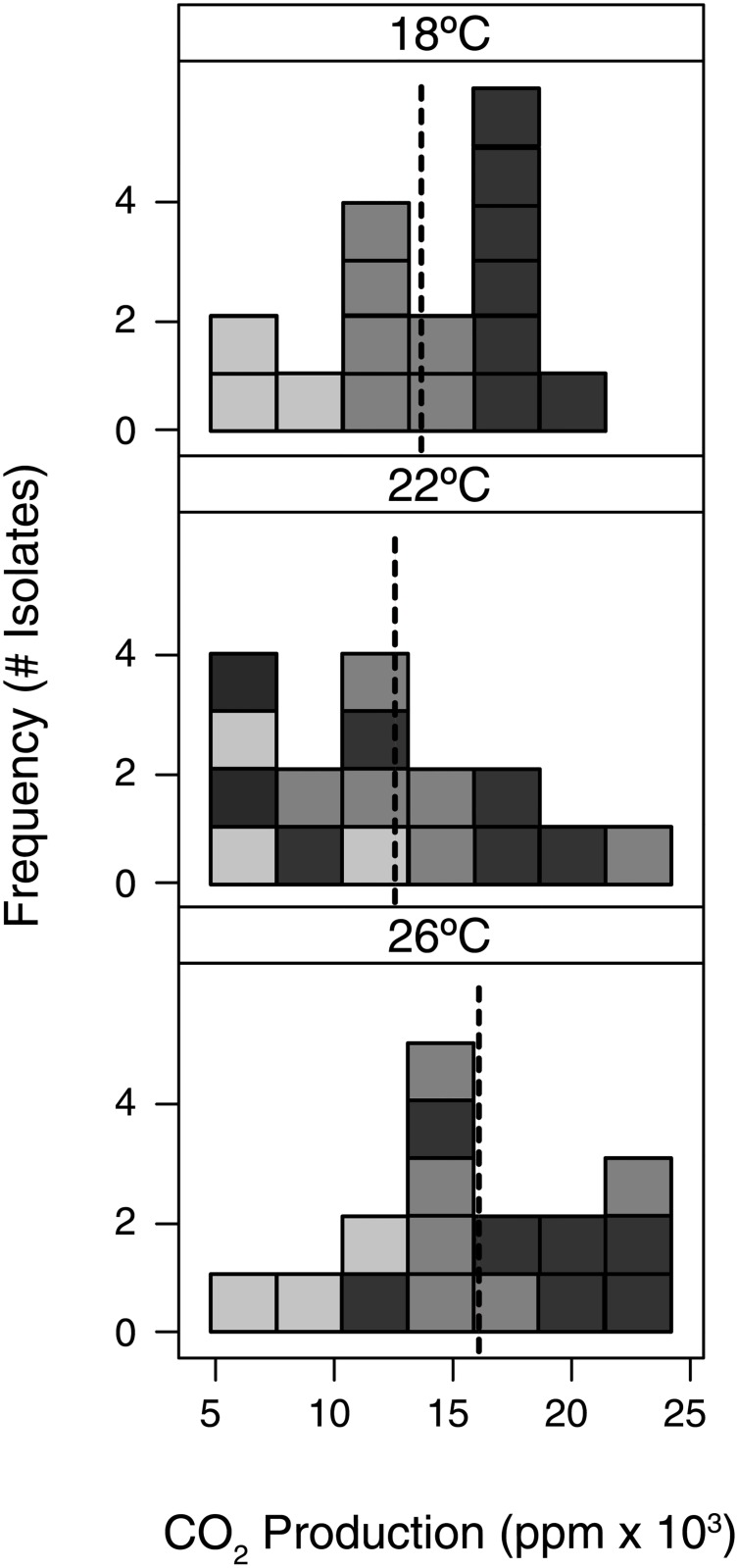
Distribution of CO_2_ production trait. The distribution of one functional trait, CO_2_ production rate, across each temperature treatment. Each box represents the cumulative CO_2_ production rate for one bacterial isolate (16 total). Isolates are shaded according to their CO_2_ production rate at 18°C, where light and dark shaded boxes are relatively low and high producers at 18°C, respectively. Vertical, dashed line indicate the mean CO_2_ production each temperature treatment.

Similar to the simple substrates, the litter bacteria varied substantively in their abilities to decompose complex leaf litter (Table 2). Decomposition rates, measured as cumulative CO_2_ production, ranged from ~5,500–23,500 ppm CO_2_ during the 10 day period (Figs 3b and 4). These values corresponded to 0.22–0.94% of litter carbon respired.

### Response to temperature

Generally, the number of substrates utilized by the isolates depended on the temperature of the assay. The median number of simple substrates used by each isolate was highest at the intermediate temperature 22°C (n = 13) and declined to 10 substrates at 18°C and 9 substrates at 26°C (S2 Fig). Mean growth rate (μ_max_) and substrate use (OD_590_) on the EcoPlates also peaked at 22°C. In addition, isolate and temperature significantly interacted to affect the mean cumulative substrate use (OD_590_; mixed-model ANOVA; Table 2).

We also observed a significant isolate and temperature interaction on CO_2_ production rates (Table 2; S3 Fig); decomposition rates were generally highest at 26°C, but the mean rate at 22°C tended to be lower than at 18°C (Fig 4).

### Phylogenetic signal

**Fig 5:**
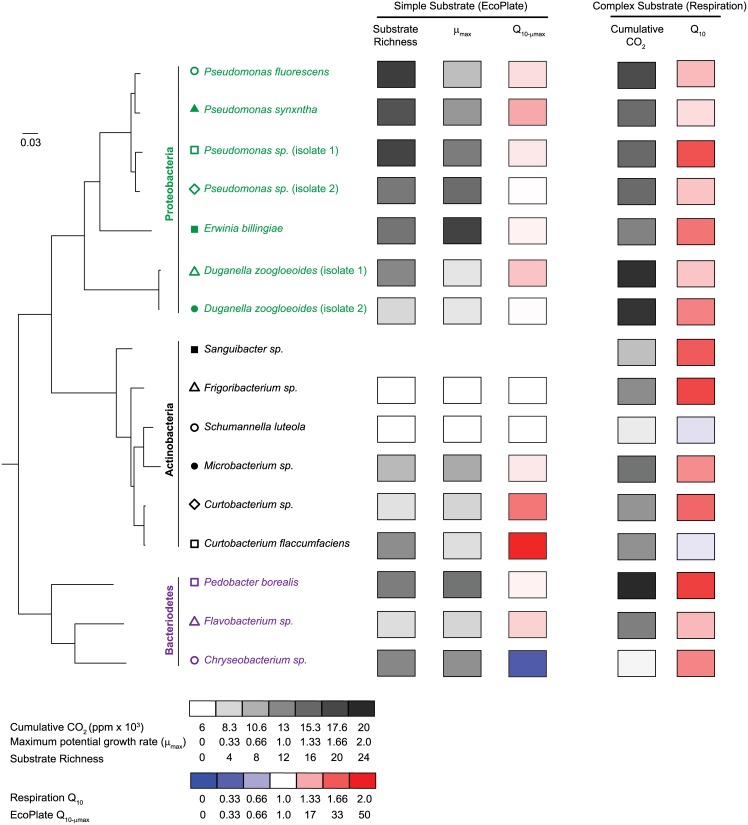
Phylogenetic tree of bacterial isolates with functional and response traits. Maximum likelihood phylogenetic tree of bacterial isolates based on the majority consensus of 100 bootstrap runs using Phylip. Isolate names shown in green, black, and purple belong to phyla Proteobacteria, Actinobacteria, and Bacteriodetes, respectively. Symbols next to each isolate name match those used in Fig 6. Plotted next to each isolate is the summary of functional and response traits for simple and complex substrates. All functional traits are based on the mean value across all temperatures and, when applicable, EcoPlate substrates.

We found that a number of the functional traits were phylogenetically conserved. The overall substrate use profile, defined as the suite of simple EcoPlate substrates used by each isolate, was correlated with phylogenetic distance (Mantel Test: rM = 0.23). This was also the case when we examined the μ_max_ values for the suite of substrates used by each isolate (Mantel Test: rM = 0.20; Table 3). Thus, isolates that were more closely related shared more similar carbon use patterns and grew at similar growth rates. In addition, the average μ_max_ across all utilized substrates was also strongly conserved among isolates (K-statistic: 0.44, p = 0.002), and was generally highest in Proteobacteria and lower in Actinobacteria (Fig 5). While the number of substrates that each isolate could utilize did not display a phylogenetic pattern (substrate richness, Fig 5), the ability to use particular substrates did; these patterns were consistent if we examined each temperature treatment individually (data not shown) or took the average of all three temperatures (Table 3). For example, mannitol was metabolized by isolates in all three phyla but the maximum potential growth rate (u_max_) was generally higher in isolates belonging to the Proteobacteria phyla. Alternatively, α-cyclodextrin was only utilized by two isolates, *Pedobacter borealis* and *Chryseobacterium sp*., both of which belonged to the phylum Bacteriodetes.

The ability to decompose the complex leaf litter also displayed a significant phylogenetic pattern (Fig 5), and this signal remained whether we examined each temperature individually (data not shown) or averaged across temperatures (K-statistic: 0.40, p = 0.001). On average, Proteobacteria produced more CO_2_ on the leaf litter than Bacteriodetes and Actinobacteria.

Unlike substrate use traits, we found little evidence of a phylogenetic pattern in the sensitivity of taxa to changes in temperature (Fig 5, Table 3). This was the case when we examined the Q_10_ of CO_2_ production rates (K-statistic: 0.042, p = 0.80; Fig 5), and the average μ_max_ or OD_590_ across all utilized Biolog substrates (K-statistic: 0.28 (p = 0.52) and 0.08 (p = 0.74), respectively). A few individual substrates did have Q_10_ values (based on both μ_max_ and OD_590_) that were phylogenetically conserved (Table 3), but these significant correlations did not appear to be related to substrate type (polymer, amino acid, etc.).

### Correlations between substrate use & temperature response

**Fig 6:**
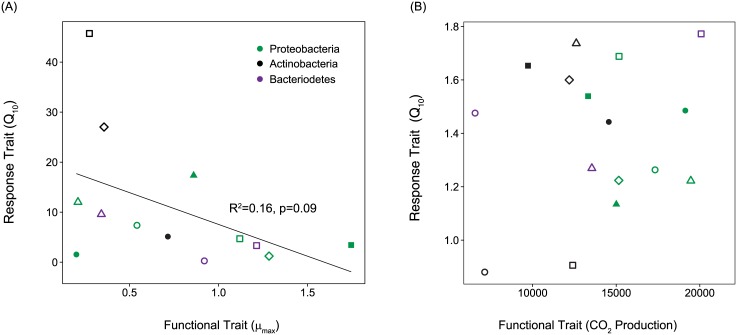
Functional and response trait correlation. Correlation between the functional traits (A) μ_max_ and (B) cumulative CO_2_ production and each trait’s sensitivity to temperature (Q_10_). Each isolate is represented by a unique symbol and corresponds to the symbols used in Fig 5. Symbols shown in green, black, and purple represent isolates belonging to the phyla Proteobacteria, Actinobacteria, and Bacteriodetes, respectively.

The functional and response traits of the litter bacteria were generally not correlated. A isolate’s average μ_max_ tended to be negatively related with Q_10-μmax_, but this pattern was not statistically significant (R^2^ = 0.16, p = 0.09; Fig 6A). We also found no correlation between a isolate’s average CO_2_ production rate and its sensitivity to temperature (Q_10_; Fig 6B). Functional and response traits were generally orthogonal to one another in a PCA ordination (S4 Fig).

## Discussion

Our co-occurring leaf litter bacteria varied substantially in their substrate use abilities. While this variation has been previously documented (as seen in [46–48]), there are surprisingly few studies that assay such parameters for isolates isolated from the same community. Although trait values revealed in a laboratory setting may not translate to real-word conditions, these data reveal an estimate of the distribution of traits in a particular community and the breadth of occupied niche space [49]. Such data can also be used to parameterize trait-based models that predict how microbial communities, and their ecosystem functions, will respond to changing environmental conditions (e.g. [50–53]).

The simple substrate assays used in our study revealed a large range in potential substrate use among leaf litter bacterial taxa. Indeed, some isolates appeared to specialize on a small number of substrates, while others metabolized a much higher number. The use of the EcoPlate system to analyze microbial traits is not without its weaknesses. Simple substrates do not cover the whole diversity of potential substrates that can be found in leaf litter, and microbial activity in the EcoPlate may not be identical to that in nature. Despite these weaknesses, we observed that isolates that utilized more EcoPlate substrates also had higher rates of CO_2_ production on complex leaf litter (R^2^ = 0.08, p = 0.05).

Changes in temperature also influenced the bacteria’s ability to utilize simple and complex carbon substrates as previously observed (reviewed in [12]). For example, u_max_ and substrate use richness of the *Chryseobacterium sp*. isolate (family Flavobacteriaceae) increased with temperature. Indeed, earlier studies demonstrated that many strains of *Chryseobacterium* can grow at temperatures as high as 37°C [54]. On the other hand, the substrate use richness of both *Curtobacterium* isolates (family Microbacteriaceae) decreased with temperature.

While warmer temperatures generally increase decomposition rates through a combination of increased enzymatic activity, metabolism, and growth (increased abundance) [2, 55, 56], we observed mixed responses to increasing temperatures. In general, the usage of simple carbon substrates (via Ecoplates) decreased with temperature, although the decomposition of complex leaf litter mildly increased with temperature. Unlike many other studies [55, 57, 58], we measured temperature response using a relatively narrow temperature range. This narrow temperature range, and focus on more labile substrates, could have hindered our ability to measure an accurate temperature effect. Other studies have demonstrated that the decomposition of labile compounds is generally less sensitive to changes in temperature [59]. Many, if not all, of the EcoPlate substrates used in this study are relatively labile compounds. In addition, it's likely that the CO_2_ produced in our microcosm assay was mineralized from labile substrates, as these are the first targets of decomposition. If this is the case, conducting similar experiments on more recalcitrant substrates will be essential to assessing microbial responses to environmental change.

As hypothesized, the ability of the leaf litter taxa to utilize substrates was partially phylogenetically conserved. This was the case whether we analyzed simple substrates (via EcoPlates) or natural, more complex, leaf litter. However, contrary to our expectation that these traits would only be finely-conserved, we observed a phylogenetic pattern across broad taxonomic lineages. For example, isolates belonging to the phyla Proteobacteria, on average, utilized more substrates and had higher decomposition rates than isolates belonging to the phyla Actinobacteria and Bacteriodetes (Fig 5). These Proteobacteria (class γ-Protebacteria and β-Proteobacteria) are generally characterized as fast growers that target lower molecular weight (more labile) compounds [60–63]. In contrast, the response of both Actinobacteria and Bacteriodetes to soil carbon substrates appears mixed [62, 63].

Unlike substrate usage, we found that temperature response did not appear to be phylogenetically conserved. This is in contrast to our hypothesis and previous studies, which have observed environmental conditions like light and moisture availability to result in the phylogenetic, clustering of microbial communities (e.g. [32, 64]). In this system in particular, we previously observed significantly conserved responses to drought and nitrogen addition in the field, at least among closely related taxa (<0.4% similar in 16S sequence [33]). However, this example also raises the possibility that the temperature response is similarly conserved at a fine genetic scale. In other words, the adaptation to minor temperature changes might evolve on shorter time scales and involve simpler traits, resulting in a relatively shallow level of conservation [35]. In this case, our selection of 16 isolates across three phyla reduced our ability to detect a phylogenetic signal at finer genetic scales (within phyla), and limits our ability to pinpoint the depth of conservation (e.g., using metric such as consenTRAIT [31]).

Contrary to our final hypothesis, we did not detect a correlation between the functional traits and temperature response of these leaf litter bacteria. A correlation between functional and response traits might indicate a physiological tradeoff, as organisms are often constrained by their ability to maximize every trait. One evolutionary consequence of physiological tradeoffs is organisms that exist along a continuum of life strategies, from stress-tolerant, slow-growing generalists to fast-growing but more susceptible specialists. For instance, Fig 1 depicts a scenario where taxa that have higher decomposition rates at 18°C respond less to increases in temperature. However, we found that the leaf litter isolates that utilized a larger set of substrates (i.e., more generalist life-strategies) were no more or less sensitive to temperature changes than isolates that utilized fewer substrates (i.e. specialist life-strategies) (Figs 4 and 6). While many of an organism’s traits are expected to be correlated (for instance, plant leaf traits [65], the mechanism driving trait linkage varies. For example, trait correlations could be due to biological or physical tradeoffs (e.g., size and nutrient uptake in phytoplankton [8]). In addition, these patterns could also be produced when the same genes influence multiple traits (i.e. pleiotropy).

Although accompanied by recognized biases, culture-based studies offer a path for characterizing the distribution of traits in a microbial community and its response to changing environmental conditions. These microbial trait distributions can also be incorporated into models that predict how climate change will influence ecosystem functioning [66–68]. As this area of research grows, future studies should determine whether the phylogenetic patterns observed here apply to other microbial communities (residing on leaf litter or other environments). Further work should also evaluate if the responses of particular taxa to environmental change vary in different ecosystems. Overall, these types of studies will improve our understanding of how functional and response traits are distributed in a microbial community, and may help predict how composition, and microbial-controlled ecosystem functions, will shift in response to future environmental change.

## Supporting information

S1 Fig Substrate use richness for each isolate. The number of substrates utilized by the bacterial isolates across the three temperatures.
(EPS)

S2 Fig Substrate use richness at each temperature. Substrate use richness for all bacterial isolates for each temperature treatment. N = 15 for each temperature treatment.
(EPS)

S3 Fig Decomposition rates for each bacterial isolate. Cumulative CO_2_ production rates for each bacterial isolate across three temperature treatments. N = 3 for each box.
(EPS)

S4 Fig Principle Coordinates Analysis (PCA) of functional and response traits. PCA ordination based on Euclidian distances of normalized functional and response traits. All functional traits are based on the mean value across all temperatures and, when applicable, EcoPlate substrates. The color of the symbols represent the phyla: Proteobacteria (green), Actinobacteria (black), or Bacteriodetes (purple). Symbols correspond to those shown in Fig 5.
(EPS)

S1 Dataset Biolog EcoPlate and decomposition data. Raw microcosm and CO_2_ production data for all isolates across three temperature treatments.
(XLSX)
